# Broadly Neutralizing Hemagglutinin Stalk-Specific Antibodies Induce Potent Phagocytosis of Immune Complexes by Neutrophils in an Fc-Dependent Manner

**DOI:** 10.1128/mBio.01624-16

**Published:** 2016-10-04

**Authors:** Caitlin E. Mullarkey, Mark J. Bailey, Diana A. Golubeva, Gene S. Tan, Raffael Nachbagauer, Wenqian He, Kyle E. Novakowski, Dawn M. Bowdish, Matthew S. Miller, Peter Palese

**Affiliations:** a Department of Microbiology, Icahn School of Medicine at Mount Sinai, New York, New York, USA; b Department of Pathology and Molecular Medicine, Institute for Infectious Disease Research, McMaster Immunology Research Centre, McMaster University, Hamilton, Ontario, Canada; c Department of Biochemistry and Biomedical Sciences, Institute for Infectious Disease Research, McMaster Immunology Research Centre, McMaster University, Hamilton, Ontario, Canada

## Abstract

Broadly neutralizing antibodies that recognize the conserved hemagglutinin (HA) stalk have emerged as exciting new biotherapeutic tools to combat seasonal and pandemic influenza viruses. Our general understanding of the mechanisms by which stalk-specific antibodies achieve protection is rapidly evolving. It has recently been demonstrated that broadly neutralizing HA stalk-specific IgG antibodies require Fc-Fcγ receptor (FcγR) interactions for optimal protection *in vivo*. Here we examine the neutrophil effector functions induced by stalk-specific antibodies. As the most abundant subset of blood leukocytes, neutrophils represent a critical innate effector cell population and serve an instrumental role in orchestrating downstream adaptive responses to influenza virus infection. Yet, the interplay of HA stalk-specific IgG, Fc-FcγR engagement, and neutrophils has remained largely uncharacterized. Using an *in vitro* assay to detect the production of reactive oxygen species (ROS), we show that human and mouse monoclonal HA stalk-specific IgG antibodies are able to induce the production of ROS by neutrophils, while HA head-specific antibodies do not. Furthermore, our results indicate that the production of ROS is dependent on Fc receptor (FcR) engagement and phagocytosis. We went on to assess the ability of monoclonal HA stalk-specific IgA antibodies to induce ROS. Consistent with our findings for monoclonal IgGs, only HA stalk-specific IgA antibodies elicited ROS production by neutrophils. This induction is dependent on the engagement of FcαR1. Taken together, our findings describe a novel FcR-dependent effector function induced by HA stalk-specific IgG and IgA antibodies, and importantly, our studies shed light on the mechanisms by which HA stalk-specific antibodies achieve protection.

## IMPORTANCE

The present study provides evidence that broadly neutralizing HA stalk-specific antibodies induce downstream Fc-mediated neutrophil effector functions. In addition to their ability to neutralize, this class of antibodies has been shown to rely on Fc-Fc receptor interactions for optimal protection *in vivo*. Curiously, neutralizing antibodies that bind the HA head domain do not require such interactions. Our findings build on these previous observations and provide a more complete picture of the relationship between stalk-specific antibodies and cells of the innate immune compartment. Furthermore, our data suggest that the ability of HA stalk-specific antibodies to mediate Fc-Fc receptor engagement is epitope dependent. Overall, this work will inform the rational design of improved influenza virus vaccines and therapeutics.

## INTRODUCTION

Influenza virus vaccines, for decades, have been the mainstay approach to combat seasonal influenza and pandemic outbreaks. While our knowledge of influenza viruses and the immune response to these viruses has grown tremendously, innovative vaccine strategies have lagged behind. With the recent discovery of broadly neutralizing antibodies (bnAbs) that recognize the conserved stalk of hemagglutinin (HA), we are closer to realizing the goal of a universal influenza virus vaccine (1, 2). Vaccination strategies that elicit high titers of bnAbs to the HA stalk have been shown to be highly protective in several animal models (3–7). Although at steady state these stalk-specific antibodies are present at low levels, reassuringly an increasing body of evidence in humans has demonstrated that exposure to antigenically diverse influenza virus strains can boost titers of bnAbs in humans (8–15). The mechanisms by which bnAbs achieve protection are fundamentally different from strain-specific antibodies that target the globular head domain. While the latter prevent binding of HA to sialic acid on the host cell receptor, stalk-specific antibodies can prevent fusion of virus and endosomal membranes, interfere with viral egress, and inhibit cleavage of HA required for maturation (16). Importantly, recent work has revealed that Fc-Fcγ receptor (FcγR) engagement is required for HA stalk-specific antibodies to achieve optimal protection *in vivo* (17). DiLillo and colleagues speculated that antibody-dependent cell-mediated cytotoxicity (ADCC) could be the FcγR-dependent mechanism responsible for protection *in vivo* (17). Given the ubiquitous expression of FcγR on innate immune cells, this led us to hypothesize that additional Fc-mediated effector functions would also be elicited by this class of antibodies.

Phagocytosis represents a critical effector function employed by immune cells to aid in pathogen clearance and antigen presentation and to initiate immune cell activation (18). Moreover, antibody-dependent cellular phagocytosis (ACDP) of immune complexes and opsonized pathogens is a potent antibody-mediated effector function. In mice, FcR-mediated phagocytosis has been shown to be important in the clearance of influenza virus in the context of a lethal virus challenge (19). While the aforementioned studies point to macrophages as being crucial for viral clearance, neutrophils can also act as professional phagocytes. Neutrophils, a subset of polymorphonuclear granulocytes (PMNs), are the most abundant white blood cell in circulation (20). As one of the first cell populations recruited to the site of infection or inflammation, neutrophils play a critical role in orchestrating an effective immune response both directly through diverse effector mechanisms and indirectly through the secretion of cytokines (20). In mouse models of influenza virus infection, optimal CD8^+^ T-cell-mediated immunity relies on the recruitment of neutrophils to the infected trachea (21). Undoubtedly, the most well-established effector function of neutrophils is their capacity to phagocytose. After neutrophils engulf pathogens into the membrane-bound phagosome, fusion of the phagosome with cytoplasmic granules leads to the formation of the phagolysosome. Once in the phagolysosome, microorganisms are exposed to antimicrobial peptides and reactive oxygen species (ROS) generated by the NADPH complex which eventually kills the pathogen (22).

While several studies have illustrated the critical role of neutrophils in limiting viral replication (23, 24), there is a paucity of data regarding the antibody-mediated effector function of neutrophils in the context of influenza virus infection. As innate immune cells, neutrophils express both activating and inhibitory FcγRs on their surface. Murine PMNs express all four FcγRs, including the activating receptors FcγRI, FcγRIII, and FcγRIV and the lone inhibitory receptor FcγRIIB (25). Human neutrophils, on the other hand, express three of the six FcγRs, including the activing receptors FcγRIIA, the inhibitory receptor FcγRIIB, and the curious FcγRIIIB which lacks a transmembrane domain (26, 27). Here we looked to unravel the Fc-mediated effector functions induced by bnAbs to the conserved HA stalk by examining the interplay between this antibody class and neutrophils. Using a panel of HA-specific murine and human antibodies, we performed an *in vitro* luminol-based assay to measure the production of ROS by neutrophils when stimulated with immune complexes. Here we show that antibodies that recognize the HA globular head domain and possess classic hemagglutination inhibition activity did not induce ROS, while HA stalk-specific antibodies potently elicited ROS. We found that ROS induction was dependent on Fc-FcR interactions and was diminished in the presence of phagocytosis inhibitors, indicating that HA stalk-specific antibodies elicit antibody-dependent cellular phagocytosis. We suggest that the preferential ability of HA stalk-specific antibodies to initiate downstream Fc-mediated effector functions is epitope dependent. Taken together, our findings reveal a previously unappreciated effector function of HA stalk-specific bnAbs and help to provide a more complete picture of the mechanisms induced by this class of antibodies.

## RESULTS

### Monoclonal HA stalk-specific antibodies induce the production of ROS by neutrophils, while antibodies recognizing the HA head domain do not.

**FIG 1:**
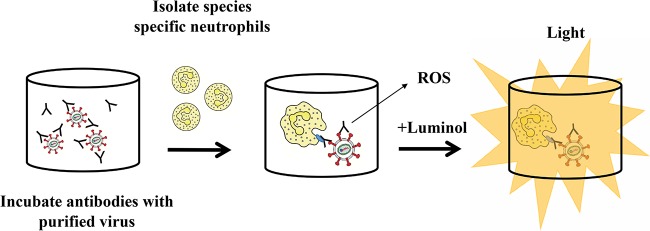
Schematic of *in vitro* luminol-based assay to measure ROS. Immune complexes were formed by incubating 5 µg/well purified influenza A viruses with 10 µg of a given antibody for 30 min at room temperature in Nunc opaque MaxiSorp 96-well plates. Neutrophils were isolated either from peripheral human blood or murine bone marrow. Prior to the addition of neutrophils, 50 µl of luminol was added to all wells. Following isolation, 50 µl of neutrophils (5 × 10^5^ cells per well) was added into the system. Luminescence (in relative light units [RLU]), resulting from the interaction of luminol with ROS, was assessed using a microplate reader. To gauge the relative ability of antibodies to induce ROS, we calculated an indexed RLU value (33), where the reference sample for all experiments was neutrophils (PMNs) in the presence of virus.

**FIG 2:**
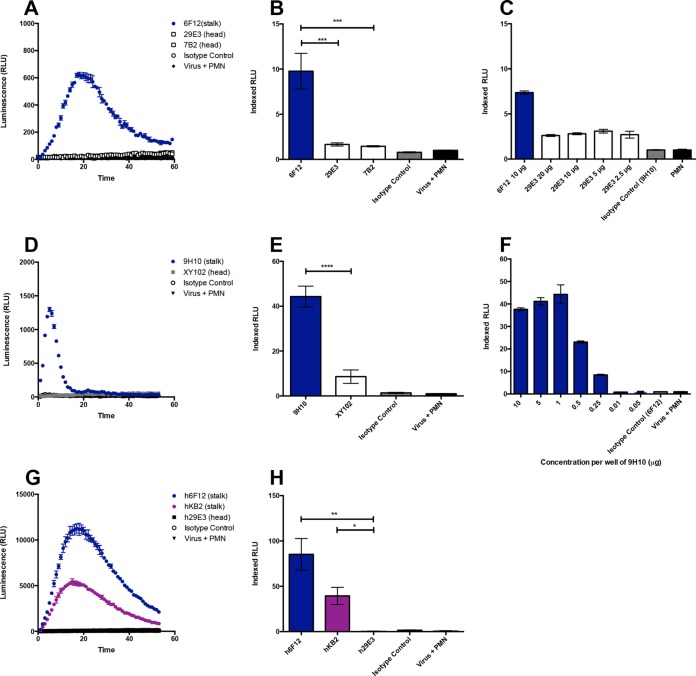
Monoclonal HA stalk-specific antibodies induce the production of ROS by neutrophils. Primary murine (A to F) or human (G and H) neutrophils (PMNs) were isolated and assessed for their ability to produce ROS in an *in vitro* luminol-based assay. Immune complexes were formed by incubating antibodies with purified H1N1 A/California/04/09 virus (A to C, G, and H) or X-31 virus (H3N2) (D to F). (A to C) The broadly neutralizing H1 stalk-specific murine antibody 6F12 and strain-specific antibodies 29E3 and 7B2 were tested for ROS induction. (D to F) The same assay was repeated using the H3 stalk-specific antibody 9H10 and head-specific antibody XY102. (G and H) Chimeric H1 broadly neutralizing stalk-specific antibodies h6F12 and hKB2 were also evaluated for their ability to induce ROS along with the strain-specific head antibody h29E3. Representative time course data for a single experiment (A, D, and G) and indexed RLU values (33) from three independent experiments (B, C, E, F, and H) are shown. Values are means ± standard errors of means (error bars). Values that are significantly different by the Kruskal-Wallis test with Dunn’s multiple comparison (B and F) or by the Mann-Whitney test (C) are indicated by bars and asterisks as follows: *, *P* < 0.05; **, *P* < 0.01; ***, *P* < 0.005; ****, *P* < 0.0001.

Broadly neutralizing stalk-specific HA antibodies have been shown to require Fc-Fcγ receptor (FcγR) interaction for optimal protection *in vivo* (17), yet the full scope of Fc-mediated effector functions elicited by these antibodies has not been fully described. To investigate the interplay between HA stalk-specific antibodies and neutrophils, we utilized an *in vitro* luminol-based assay to measure the production of ROS by neutrophils when stimulated with immune complexes. The production of various species of oxygen radicals by neutrophils can be induced by soluble and particulate agonists, and these ROS both directly and indirectly aid in pathogen clearance (28). For these studies, we used a panel of mouse monoclonal HA-specific antibodies that have been previously described by our group (29–32). The antibody, 6F12, binds to the stalk domain of H1 viruses, while the antibodies 7B2 and 293E are specific to the head domain of A/California/04/09 (Cal09) virus. Immune complexes were formed by incubating 5 µg/well of purified Cal09 virus with 10 µg of mouse monoclonal antibody. These complexes were used to stimulate murine neutrophils isolated from bone marrow. Finally, to allow for the detection of respiratory burst activity, luminol was added to the system. In this setup, luminol reacts with ROS generated by neutrophils to produce an excited state intermediate that emits light as it relaxes to the ground state (Fig. 1). Furthermore, luminol is capable of crossing biological membranes allowing for the detection of oxygen species originating from the phagosome. When murine neutrophils were incubated with immune complexes formed using the HA stalk-specific antibody 6F12, we observed a potent induction of ROS (Fig. 2A and B). To gauge the relative ability of monoclonal antibodies to induce ROS production, results were indexed according to a baseline activation of neutrophils by purified virus, as described in Materials and Methods. A similar approach has previously been reported (33). Interestingly, antibodies recognizing the head domain of HA did not induce ROS production over baseline levels (Fig. 2A and B). This observation held true even when a high concentration of head-specific antibody was used (Fig. 2C).

To ensure that this phenomenon was not subtype specific, we repeated the experiment using the influenza A virus X-31 (H3N2). These studies used the broadly neutralizing stalk antibody 9H10 as well as the H3 head-specific neutralizing antibody XY102 (34, 35). Consistent with our previous result, we observed that immune complexes formed using the HA stalk-specific antibody 9H10 potently induced ROS production (Fig. 2D and E). Immune complex formation and the induction of ROS occurred in a dose-dependent manner (Fig. 2F). While XY102 induced ROS production to a low level, this induction was fivefold lower than that observed for 9H10 (Fig. 2E).

To confirm whether these findings were relevant in the context of human samples, we utilized chimeric IgG antibodies previously described by our group (36). Briefly, these chimeric antibodies were generated by cloning the variable regions of the H1 stalk-binding antibodies 6F12 and KB2 and the HA head-specific antibody 29E3 into a human IgG1 backbone (h6F12, hKB2, and h29E3). These antibodies were expressed recombinantly in HEK 293T cells. Human neutrophils were isolated from peripheral blood, and the assay was performed using immune complexes formed with chimeric antibodies. Consistent with our findings using a murine system, we observed that only HA stalk-specific antibodies led to the generation of ROS (Fig. 2G and H). The chimeric head-specific antibody h29E3 failed to induce ROS by human neutrophils (Fig. 2G and H). Therefore, only immune complexes formed by HA stalk-specific antibodies were capable of potently stimulating respiratory burst activity by neutrophils.

### The production of ROS by monoclonal IgG antibodies is dependent on Fc-FcγR interactions.

**FIG 3:**
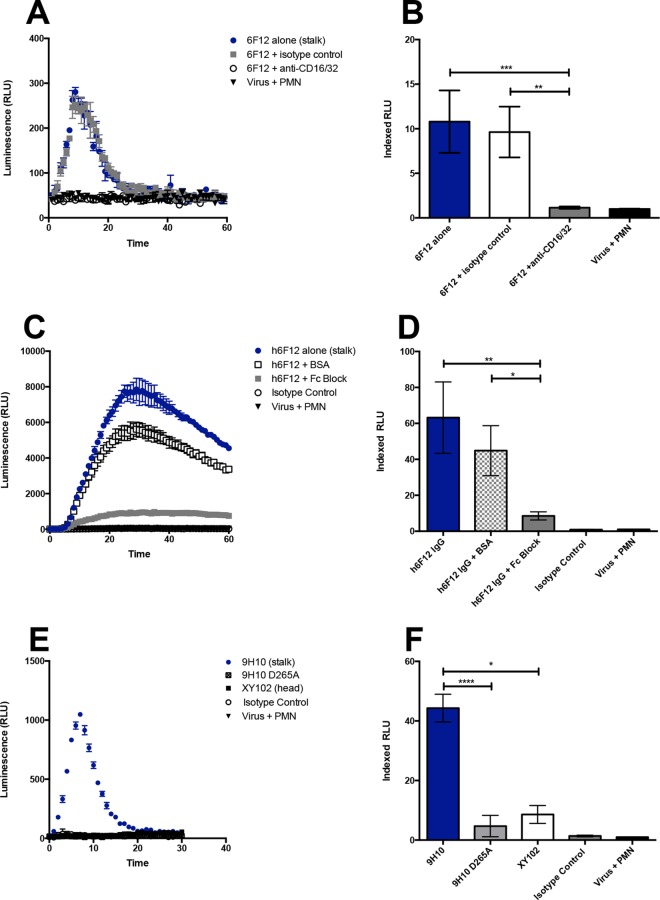
The production of ROS by monoclonal IgG antibodies is dependent on Fc-FcγR interactions. The dependence of ROS production on Fc-FcR interactions was probed either by incubating neutrophils with FcR blocking antibodies prior to addition in the assay (A to D) or engineering antibodies with a D265A mutation to ablate binding to FcγRs (E and F). The ability of the murine H1 broadly neutralizing stalk-specific antibody 6F12 to induce ROS (A and B) and the chimeric human antibody h6F12 (C and D) were assessed in the presence of Fc blocking antibodies. (E and F) The murine H3 broadly neutralizing stalk-specific antibody 9H10 engineered with a D265A mutation was examined for its ability to induce ROS. Luminol assays using this mutant were performed in parallel with the experiments shown in Fig. 2C and D, and the controls have been duplicated for ease of comparison. (A, C, and E) Representative time course from a single experiment. (B, D, and F) Indexed RLU values (33) pooled from three independent experiments. Means ± standard errors of means (error bars) are shown. Values that are significantly different by the Kruskal-Wallis test with Dunn’s multiple comparison are indicated by bars and asterisks as follows: *, *P* < 0.05; **, *P* < 0.01; ***, *P* < 0.005; ****, *P* < 0.0001.

As a critical innate effector cell population, neutrophils express both activating and inhibitory Fcγ receptors. Murine neutrophils express all FcγRs (25); however, expression of the activating receptor FcγRI has been shown to be low at steady state (37). On the other hand, human neutrophils constitutively express only three of the six FcγRs, FcγRIIA, FcγRIIB, and FcγRIIIB (26, 27). Of these three FcγRs, only FcγRIIA is an activating receptor. We assessed the dependence of ROS production on Fc-FcγR interactions in two ways. First, we blocked FcγRs on the surfaces of neutrophils by incubating cells with commercially available antibodies prior to stimulation with immune complexes. Incubation of murine neutrophils with anti-CD32/CD16 abrogated the induction of ROS by 6F12 (Fig. 3A) and led to a significant decrease in indexed relative light unit (RLU) values (Fig. 3B). Similarly, incubation of human neutrophils with Fc block (BD Pharmingen) ablated the ability of the human 6F12 (h6F12) to elicit ROS production (Fig. 3C and D). In addition to blocking FcγRs on the surfaces of neutrophils, we engineered a variant of 9H10 that lacks the ability to bind FcγRs. Replacement of aspartic acid with an alanine at position 265 (D265A) in the constant heavy chain has been shown to result in a complete loss of binding of IgG2a and IgG2b to all four classes of FcγRs (38). Compared to the wild-type (WT) 9H10 antibody, the D265A mutant failed to induce ROS by murine neutrophils (Fig. 3E and F). Taken together, these data demonstrate that Fc-FcγR interactions are required for the respiratory burst activity elicited by stalk-specific antibodies.

### Inhibition of phagocytosis ablates ROS induction by monoclonal IgG antibodies.

**FIG 4:**
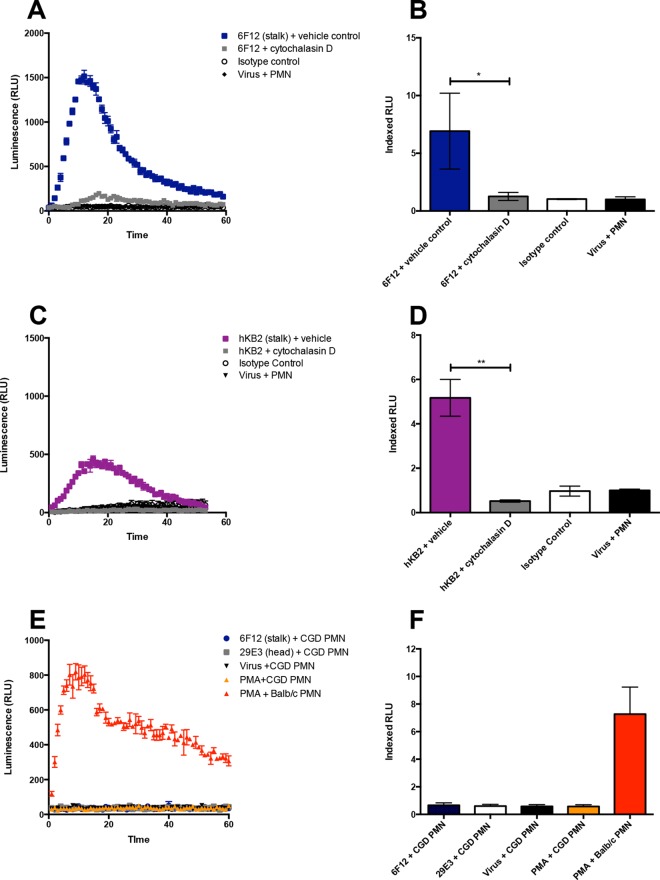
Inhibition of phagocytosis ablates ROS induction by monoclonal IgG antibodies. *In vitro* luminol-based assays were performed using murine (A and B) or human (C and D) neutrophils incubated with cytochalasin D prior to addition to the assay. (E and F) To confirm activation of NADPH oxidase by murine monoclonal antibodies, neutrophils were isolated from B6.129S-*Cybb*^tm1din^/J mice which are a model for chronic granulomatous disease (CGD). (A, C, and E) Representative time course from a single experiment. (B, D, and F) Indexed RLU values (33) pooled from three independent experiments. Means ± standard errors of means are shown. Values that are significantly different by the Mann-Whitney test are indicated by bars and asterisks as follows: *, *P* < 0.05; **, *P* < 0.01.

We next sought to determine the role of phagocytosis on respiratory burst induction. Phagocytosis represents a well-established effector mechanism utilized by neutrophils to eliminate pathogens (20). To test whether phagocytosis was required for ROS induction, we relied on cytochalasin D, a potent inhibitor of actin polymerization. Murine and human neutrophils were incubated with 10 µg/ml of cytochalasin D prior to stimulation with immune complexes. Inhibition of phagocytosis ablated the induction of ROS by the murine HA stalk-specific antibody 6F12 (Fig. 4A) and the chimeric hKB2 antibody (Fig. 4C), again leading to a significant decrease in indexed RLU values (Fig. 4B and D, respectively). We additionally probed whether the induction of ROS required phagocytosis by coating purified virus on the wells of 96-well plates, instead of allowing immune complexes to form in suspension. We were unable to observe ROS production when the *in vitro* assay was performed in this manner (data not shown). Chronic granulomatous disease is a disorder characterized by defective phagocytic respiratory burst activity. Our data thus far suggest that HA stalk-specific antibodies stimulate NADPH oxidase activity in an Fc-dependent manner. To test this hypothesis, we utilized mice with a null allele for the 91-kDa subunit of the oxidase cytochrome B. These mice lack phagocytic respiratory burst activity and are used as a model for chronic granulomatous disease. As expected, neutrophils isolated from B6.129S-*Cybb*^tm1din^/J mice did not generate ROS when stimulated with immune complexes formed with either 6F12 or 29E3 antibody (Fig. 4E and F). These data confirm that the ROS species signature elicited by HA stalk-specific antibodies represents activation of NADPH oxidase and phagocytic respiratory burst activity.

### Chimeric human stalk-specific IgAs induce ROS production through engagement of FcαR1.

**FIG 5:**
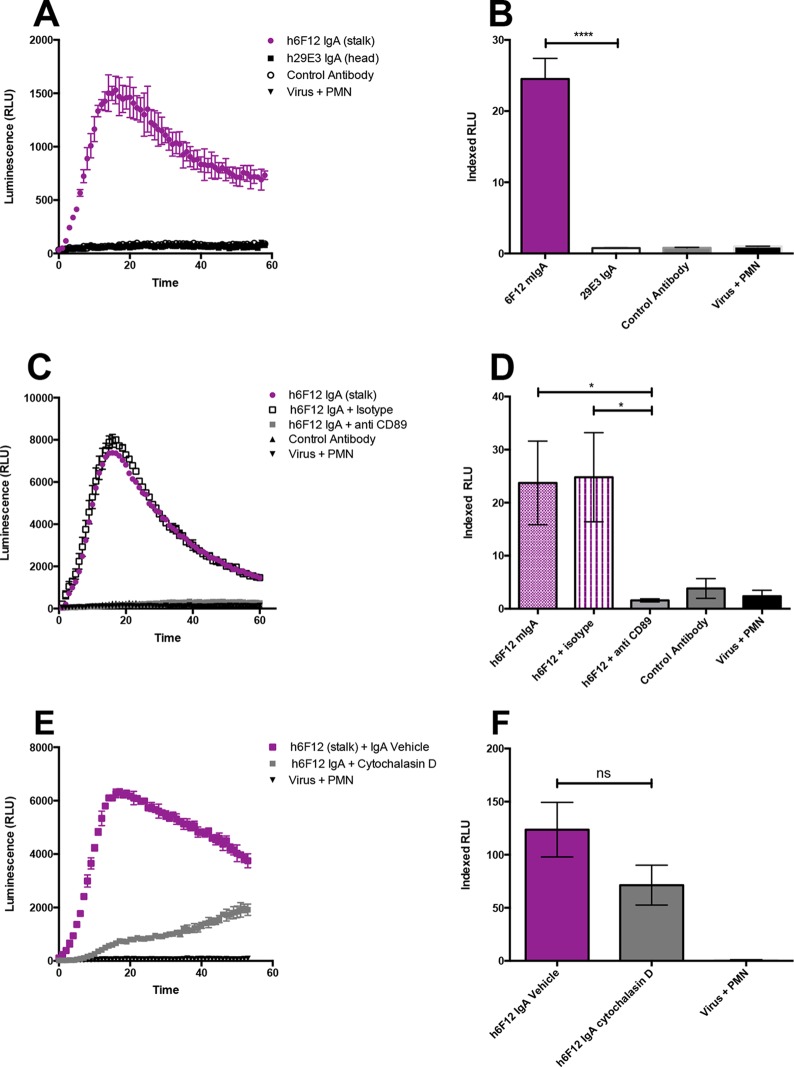
Chimeric human stalk-specific IgAs induce ROS production through engagement of FcαR1. Chimeric IgA antibodies were generated by cloning the variable regions of the H1 broadly neutralizing stalk-specific antibody 6F12 and strain-specific head antibody 29E3 into a human IgA backbone. (A and B) Chimeric IgA antibodies were assessed for ROS induction in a luminol-based assay using human neutrophils and immune complexes formed with Cal09. (C and D) Neutrophils were incubated with anti-CD89 (FcαR1) prior to the addition to the assay. (E and F) Luminol assays were repeated using neutrophils incubated with cytochalasin D prior to stimulation with immune complexes. (A, C, and E) Representative time course from a single experiment. (B, D, and F) Indexed RLU values (33) pooled from three independent experiments. Means ± standard errors of means are shown. Values that are significantly different by Student’s *t* test (B) and Kruskal-Wallis test with Dunn’s multiple comparison (D) are indicated by bars and asterisks as follows: *, *P* < 0.05; ****, *P* < 0.0001. Values that are not significant (ns) are also indicated.

IgA is the most prevalent antibody at mucosal sites, and as such, it plays a critical role in protection against respiratory pathogens such as influenza viruses (39). Our group has previously reported that B cells producing broadly neutralizing antibodies of the IgA isotype are widespread (36). Having observed that HA stalk-specific IgG antibodies induce ROS by neutrophils, we went on to examine whether the same phenomenon could be observed using both HA stalk-specific and head-binding monomeric IgA (mIgA) antibodies. Chimeric IgA antibodies were generated by cloning the variable regions of the H1 broadly neutralizing stalk-specific antibody 6F12 and strain-specific head antibody 29E3 into a human IgA backbone. Consistent with our results for chimeric IgG antibodies, only h6F12 was able to induce the production of ROS by human neutrophils (Fig. 5A and B). The IgA-triggering Fc receptor, FcαRI (CD89) is constitutively expressed on neutrophils; therefore, we turned our attention to investigate whether the generation of ROS by mIgA requires FcαRI. To achieve this, human neutrophils were incubated with an anti-CD89 antibody prior to stimulation with immune complexes. In line with our earlier findings, blocking the IgA receptor FcαRI abolished ROS production and led to a significant reduction in indexed RLU values (Fig. 5C and D). These studies were performed using only human neutrophils, as mice lack a FcαRI ortholog. Finally, we went on to probe the role of phagocytosis on respiratory burst activity. As before, neutrophils were incubated with cytochalasin D prior to addition to the assay. While the induction of ROS was delayed when neutrophils were incubated with cytochalasin D, interestingly, ROS production was not entirely ablated (Fig. 5E). This observation held true even when increasing concentrations of cytochalasin D were utilized (data not shown). Therefore, while we observed a decrease in ROS production when phagocytosis was inhibited, no significant difference in indexed RLU values were observed between h6F12 IgA plus vehicle and h6F12 plus cytochalasin D (Fig. 5F). These data suggest that, in addition to phagocytosis, HA stalk-specific IgA antibodies are able to induce ROS by other Fc-related mechanisms. Consequently, our data show that both HA stalk-specific IgG and IgA antibodies elicit respiratory burst activity of human neutrophils.

### Murine and human stalk-specific IgG antibodies activate the antibody-dependent cellular phagocytosis pathway in an epitope-specific manner.

Our previous results demonstrated that murine and human HA stalk-specific IgG antibodies led to respiratory burst activity of neutrophils in a manner that is dependent on both Fc-FcγR engagement and phagocytosis. To confirm that these IgG antibodies are capable of eliciting antibody-dependent cellular phagocytosis (ADCP), we relied on a commercially available ADCP assay whereby A549 cells infected with influenza A viruses express HA on the cell surface. These conditions mimic how HA would be presented in the context of a natural infection. Following the addition of HA-specific antibodies to infected cells, a reporter cell line that stably expresses human FcγRIIA is added into this system. This allows for the quantification of ADCP induction by luciferase expression.

**FIG 6:**
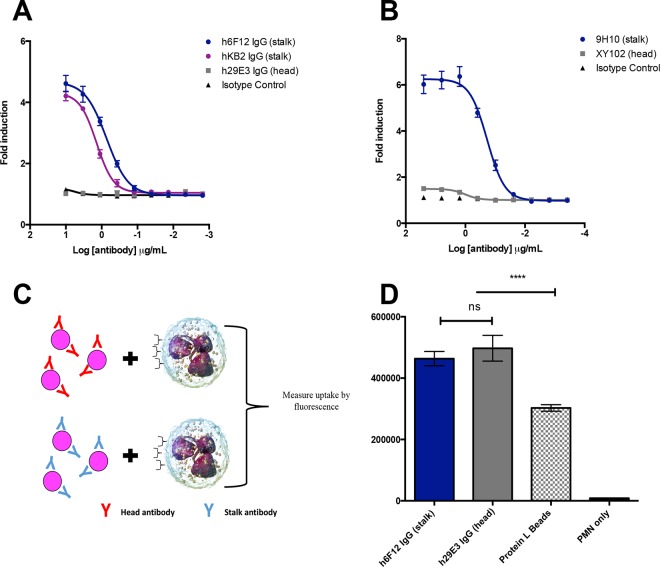
Murine and human stalk-specific IgG antibodies activate the antibody-dependent cellular phagocytosis (ADCP) pathway in an epitope-specific manner. (A and B) ADCP reporter assays were performed on Cal09-infected (A) or X-31-infected (B) A549 cells using a panel of human (A) or murine (B) monoclonal antibodies. (A) The broadly neutralizing stalk-specific antibodies h6F12 and hKB2 were assessed for their ability to activate FcγRIIA. (B) The same assay was repeated using the broadly neutralizing HA stalk binding antibody 9H10 and head binding antibody XY102. The means and standard errors of means are shown for three independent experiments. (C) Bead-based phagocytosis assays were performed using fluorescent microspheres sequentially coated with protein L, followed by either the HA stalk-specific antibody h6F12 or the strain-specific head antibody h29E3. Preparation of the beads in this way leaves the antibody Fc region accessible. (D) Freshly isolated human neutrophils were incubated with microspheres. Following incubation, unbound microspheres were removed by centrifugation and washing; uptake was measured by fluorescence. Means and standard errors of means are shown. Data are representative of three independent experiments (*n* = 8 donors in total). Values that are significantly different (*P* < 0.0001) by Kruskal-Wallis test with Dunn’s multiple comparison are indicated by the bar and four asterisks. Values that are not significant (ns) are also indicated.

Cells infected with A/Netherlands/602/09 (H1N1) were incubated with the chimeric IgG antibodies h6F12, hKB2, and h29E3 at doses ranging from 10 to 0.013 µg/ml. As expected, h6F12 and hKB2 induced ADCP, whereas h29E3 did not (Fig. 6A). Given that human FcγRIIA has previously been shown to bind murine IgG2a and IgG2b antibodies (40), we used this reporter system to additionally test the murine HA stalk-specific antibody 9H10 and the HA head-specific antibody XY102. Cells were infected with X-31 (H3N2) and incubated with antibody concentrations from 25 to 0.000381 µg/ml. Again, we observed that while XY102 failed to activate the reporter cell line, 9H10 potently induced ADCP (Fig. 6B).

The results from the studies presented here support previous work that broadly neutralizing HA stalk-specific antibodies readily induce FcR-mediated effector functions, while HA head-specific antibodies do not (17). However, thus far, the ability of HA monoclonal antibodies to induce downstream Fc-mediated effector function has been assessed in an epitope-dependent fashion. It is possible that binding of each antibody class to its respective epitope affects the accessibility of the Fc region. To dissect whether the ability of HA stalk-specific antibodies to elicit Fc effector functions is an intrinsic property of this antibody class or in fact dependent on the epitope, we made use of a microsphere phagocytosis assay. Yellow fluorescent carboxylate microspheres were coated first with recombinant protein L, followed by adsorption of either h6F12 or h29E3. Protein L binds immunoglobulins via the light chain, and therefore, coating the microspheres in this way leaves the Fc region of each immunoglobulin accessible (Fig. 6C). Human neutrophils were isolated from peripheral blood and incubated with coated microspheres. We compared the internalization of microspheres coated with h6F12 and h29E3 to microspheres coated with protein L only. While neutrophils internalized microspheres coated with protein L, a significant enhancement of phagocytosis was observed when microspheres were coated with h6F12 and h29E3 (Fig. 6D). No significant differences were observed between h6F12- or h29E3-coated beads. Taken together, these results suggest that the differential ability of HA stalk- and head-specific antibodies to mediate Fc effector functions is epitope dependent.

## DISCUSSION

The identification of broadly neutralizing antibodies that recognize the conserved HA stalk has reinvigorated and energized efforts to develop a universal influenza virus vaccine. Multiple strategies are currently being explored to achieve high and sustained titers of these antibodies, including the utilization of chimeric hemagglutinin constructs (31), “headless” hemagglutinins (41), and nanoparticles (42–44). In looking toward moving these vaccine regimens into the clinic, it is vital to gain a better understanding of the mechanisms by which this class of antibodies achieves protection. Recent work from the Ravetch laboratory has put forth an interesting paradigm whereby broadly neutralizing HA stalk-specific monoclonal antibodies (MAbs) require Fc-FcγR interactions to mediate optimal protection *in vivo*, yet strain-specific anti-HA head MAbs do not (17). More recently, this requirement for Fc-FcγR engagement has been extended not only to HA-specific antibodies but also to broadly neutralizing neuraminidase antibodies (45). Together these data suggest that *in vivo* protection achieved by broadly reactive antibodies is dependent on the ability of these immunoglobulins to initiate downstream Fc effector functions. The underlying mechanistic reasons for these inherent differences in strain-specific and broadly reactive antibodies remain elusive, but it is an area of intense study. The ability of antibodies to induce antibody-dependent cell-mediated cytotoxicity (ADCC) has emerged as an important effector function not only for influenza but also for HIV and cancer. Although many innate immune cells express the appropriate FcγRs to mediate ADCC, only a small subset of these cells can act as professional phagocytes. In this study, we examined the ability of HA stalk-specific antibodies to mediate antibody-dependent cellular phagocytosis (ADCP) by neutrophils. Although perhaps less well studied compared to ADCC, Fc-mediated phagocytosis has been shown to significantly contribute to the clearance of influenza virus infection in mouse models (19). Furthermore, sera from healthy human donors have also been found to mediate HA-specific ADCP (46).

Using an *in vitro* luminol-based assay to measure the production of ROS, we systematically tested a panel of HA-specific human and murine monoclonal antibodies. While monocytic cell lines can be utilized in phagocytosis assays, an important strength of our experiments is the use of primary neutrophils (both murine and human) which express the full complement of Fc receptors in a physiological context. Consistent with *in vivo* data, we found that broadly neutralizing HA stalk-specific antibodies potently induce ROS, while strain-specific antibodies that recognize the HA head domain do not. We went on further to demonstrate that ROS production is dependent on both FcγR engagement and phagocytosis for monoclonal IgG antibodies. Seeing as IgA plays a crucial role at mucosal surfaces in protecting against respiratory pathogens such as influenza viruses, we additionally examined the ability of HA head- and stalk-specific antibodies to induce ROS. In line with our findings for IgG MAbs, only HA stalk-specific IgA antibodies induced ROS in a CD89-dependent fashion. Unexpectedly, we observed that ROS induction was diminished but not entirely ablated in the presence of cytochalasin D. These data suggest that HA stalk-specific IgA antibodies could elicit additional Fc-mediated effector functions by neutrophils in addition to ADCP. Future work will be required to understand whether HA stalk-specific IgA antibodies induce unique effector functions of neutrophils.

The data presented here support multiple lines of evidence that HA stalk-specific antibodies readily mediate Fc effector functions, while HA strain-specific antibodies do not. While it has been suggested that *in vivo*, antibodies possess unique glycoforms that potentiate activating Fc-FcR interactions (47), this does not fully explain our results, as chimeric human antibodies were produced recombinantly and possess identical Fc regions. To shed light on this curious dichotomy, we assessed whether the epitope recognized by each respective antibody class influences Fc-FcR interactions. Importantly, our data demonstrate that when epitope dependence is removed, the difference in the ability of HA head- and stalk-specific antibodies to elicit phagocytosis is lost. These data firmly establish that ADCP is not an intrinsic property of broadly neutralizing stalk-specific antibodies. Precisely why HA stalk-specific antibodies, which bind proximally to the viral membrane, elicit Fc-mediated effector function is not fully understood. It may be the case that HA binding to sialic acid on the surface of an immune cell helps to stabilize the immunological synapse and thus potentiates Fc clustering and downstream signaling. This binding is intact when antibodies are bound to the stalk, but antibodies recognizing the globular head could interfere with this interaction. Further studies are needed to dissect the molecular mechanisms responsible for these observations.

While neutralization is often considered to be the primary function of antibodies in antiviral immunity, there is an increasing body of evidence highlighting a vital role for Fc-related effector functions not only in the influenza field but also in the cancer and HIV fields (48–52). Here we identify a novel Fc-mediated effector function of neutrophils induced by HA stalk-specific antibodies. We went on to assess the contribution of neutrophils in the context of passive transfer *in vivo* using a lethal murine challenge model. While depletion of neutrophils did not yield an overt phenotype (data not shown), this does not preclude the involvement of this cell population in the protection achieved by HA stalk-specific antibodies. Given that this class of antibodies exhibits multiple mechanisms to achieve protection *in vivo*, it is difficult to separate out the relative contribution of each discrete cell population. Overall, our work helps to provide insight on the mechanisms elicited by broadly neutralizing HA stalk-specific antibodies.

## MATERIALS AND METHODS

### Cells and viruses.

Adenocarcinoma human alveolar basal epithelial cells (A549) and Madin-Darby canine kidney (MDCK) cells were grown in Dulbecco’s modified Eagle’s medium (Gibco) and supplemented with 10% fetal bovine serum and 100 U/ml of penicillin and streptomycin. X-31 is a reassortant virus with the six internal genes of PR8 virus and the hemagglutinin (HA) and neuraminidase (NA) of A/Hong Kong/1/68 virus (H3N2). Two pandemic H1N1 viruses were used in this study. A/California/04/09 was grown in MDCK cells and purified over a 30% sucrose cushion. Virus pellets were resuspended in phosphate-buffered saline (PBS), and protein content was quantified using the Bradford protein assay. A/Netherlands/602/09 was grown in the allantoic fluid of 10-day-old embryonated chicken eggs.

### Antibodies.

XY102, 6F12, KB2, 9H10, and 29E3 have been previously described (29–32, 34). Chimeric IgG and IgA antibodies were generated as previously described (36). Briefly, the variable regions of the group 1 HA stalk-binding monoclonal antibodies 6F12 and KB2 were cloned into human IgG and IgA backbones (pFUSE; InvivoGen). An identical strategy was used for 29E3, a strain-specific antibody that recognizes the head domain of A/California/04/09 HA.

### Antibody purification.

All mouse antibodies described herein were produced from hybridoma cultures. Supernatants from hybridoma cultures were collected, clarified by centrifugation, and filtered prior to purification. IgG antibodies were purified by gravity flow columns containing G Sepharose 4 Fast Flow (GE Healthcare). Supernatants were run over the column twice followed by a subsequent wash step with PBS. Antibodies were eluted with 45 ml of 0.1 M glycine-HCl (pH 2.7), and the eluent was neutralized with 5 ml of 2 M Tris-HCl buffer (pH 10). IgA antibodies were purified in a similar fashion using peptide M/Sepharose resin (InvivoGen). Antibodies were subsequently concentrated and dialyzed against PBS using Amicon ultracentrifugal filter units (Millipore) with a 30-kDa molecular mass cutoff.

### Animals.

All animal protocols were reviewed and approved by the Mount Sinai Institutional Animal Care and Use Committee (IACUC). Six- to 8-week-old female BALB/c mice were purchased from Jackson Laboratory and housed under specific-pathogen-free conditions. Age-matched B6.129S-*Cybb*^tm1Din^/J (also known as gp91 phox−) mice were purchased from Jackson Laboratory. These mice possess a null allele for the 91-kDa subunit of oxidase cytochrome B and are a model for chronic granulomatous disease (CGD).

### Murine neutrophil isolation.

Murine neutrophils were isolated from bone marrow extracted from harvested femurs and tibias of uninfected naive mice using density gradient centrifugation. A gradient was created by layering 3 ml of Histopaque 1077 over 3 ml of Histopaque 1119. Bone marrow was resuspended in 1 ml of Hanks buffered saline solution (HBSS), and the cell suspension was overlaid on top of the Histopaque 1077. Samples were centrifuged for 30 min at 2,000 rpm at 25°C. Neutrophils were collected at the interface of the Histopaque 1119 and 1077 layers. Cells were washed twice in HBSS, and viability was assessed by trypan blue exclusion. Neutrophils were resuspended to a final concentration of 1 × 10^7^ cells/ml.

### Human neutrophil isolation.

Peripheral blood samples were collected from healthy volunteers who provided informed consent. Studies were approved by the Hamilton Integrated Research Ethics Board. Blood was collected in EDTA collection tubes, and whole blood was layered over a gradient of Histopaque 1077/1119 as described above. Samples were centrifuged for 30 min at 2,000 rpm at 25°C. Neutrophils were collected at the interface of the Histopaque 1119 and 1077 layers. Cells were washed twice in HBSS, and viability was assessed by trypan blue exclusion. Neutrophils were resuspended to a final concentration of 1 × 10^7^ cells/ml.

### Luminol-based assay.

A suspension-based assay to detect reactive oxygen species was adapted from a similar method (33). Briefly, immune complexes were formed by incubating 5 µg/well of purified virus with 10 µg of a given antibody for 30 min at room temperature in Nunc opaque MaxiSorp 96-well plates. Species appropriate neutrophils were isolated as described above. Prior to the addition of neutrophils, luminol (Sigma) was added to all wells. Following the final wash step, 50 µl of neutrophils (5 × 10^5^ cells per well) was added. Luminescence (in relative light units [RLU]) resulting from interaction of luminol with reactive oxygen species was assessed using a Filter Max F2 microplate reader. Neutrophils spiked with 3 to 5 µl of phorbol myristate acetate (PMA) at a concentration of 0.1 mg/ml served as a positive control. Kinetic measurements were taken at 1-min intervals for 1 h. To investigate the roles of Fc signaling and phagocytosis, neutrophils were incubated with Fc block or cytochalasin D prior to the addition of cells to the assay. Fc blocking antibodies and cytochalasin D were used at a final concentration of 10 µg/ml.

### Data analysis.

To compare the relative ability of antibody groups to induce reactive oxygen species, an indexed RLU was calculated as follows (33): indexed RLU = absolute maximum RLU of test sample/absolute maximum of reference sample.

For all experiments, neutrophils incubated in the presence of virus served as the reference sample. This condition provides a baseline measurement for respiratory burst activity induced by virus.

### Microsphere-based phagocytosis assay.

Yellow fluorescent carboxylate microspheres (0.5 µm; Polysciences, Warrington, PA) were coated with protein L, followed by either HA head- or stalk-specific monoclonal antibodies. This was achieved by sequentially incubating microspheres first with 750 µg of protein L for 4 h at room temperature and then overnight with 300 µg of the monoclonal antibody with washing steps in between incubation steps. Following the last incubation, beads were pelleted at 12,000 rpm for 10 min and washed with PBS for 30 min at room temperature with gentle mixing. Supernatants were harvested for protein determination. Beads were resuspended in a final volume of 500 µl. Primary human neutrophils were freshly isolated, and microspheres were added to aliquots of neutrophils at a ratio of 500 beads per cell. The tubes were then incubated at 37°C for 15 min with gentle shaking. At the completion of incubation, cells were centrifuged for 10 min at 2,000 rpm. The supernatant was removed from each tube, and cells were washed two times with PBS to remove all unbound microspheres. Samples were added in triplicate to a black 96-well plate, and fluorescence was measured on a SpectraMax i3 plate reader (Molecular Devices) at an excitation wavelength of 526 nm and emission wavelength of 555 nm (Ex_526_/Em_555_). Data were reported in relative fluorescent units (RFU).

### Antibody-dependent cellular phagocytosis (ADCP) cell-based assay.

A549 cells were seeded on a 96-well white bottom plate, and 24 h later, cells were infected with influenza A viruses at a multiplicity of infection (MOI) of 3 without trypsin to avoid multicycle replication. At 12 to 16 h postinfection, the inoculum was removed and replaced with assay buffer (RPMI [Gibco] with 4% low IgG serum) followed by the addition of serial dilutions of monoclonal antibodies. Antibodies and infected cells were incubated at 37°C for 30 min. At the completion of incubation, Jurkat effector cells (Promega) stably expressing FcγRIIA were resuspended in assay buffer and added to plates at a concentration of 1.5 × 10^4^ cells per well. Plates were incubated for 6 h at 37°C before the addition of Bio-Glo luciferase assay reagent (Promega). Luminescence was quantified using a Synergy 4 (Bio-Tek) plate reader. Fold induction was calculated as follows: (RLU_induced_ − RLU_background_)/(RLU_uninduced_ − RLU_background_). Curve fitting was performed with GraphPad Prism v6 software using a “log(agonist) versus normalized response variable slope (four-parameter) analysis.”

### Statistics.

Statistical analyses were performed using GraphPad Prism v6 (GraphPad Software, San Diego, CA). Data were assessed for normality using a Shapiro-Wilk test, and the appropriate statistical test was applied as indicated in figure legends. A *P* value of <0.05 was considered statistically significant throughout.
